# Grazing exclusion alters soil methane flux and methanotrophic and methanogenic communities in alpine meadows on the Qinghai–Tibet Plateau

**DOI:** 10.3389/fmicb.2023.1293720

**Published:** 2023-12-18

**Authors:** Shilin Wang, Xindong Chen, Wen Li, Wenlong Gong, Zhengwen Wang, Wenxia Cao

**Affiliations:** ^1^Key Laboratory of Grassland Ecosystem, Ministry of Education, College of Pratacultural Science, Gansu Agricultural University, Lanzhou, China; ^2^Key Laboratory of Development of Forage Germplasm in the Qinghai-Tibetan Plateau of Qinghai Province, Qinghai Academy of Animal Science and Veterinary Medicine of Qinghai University, Xining, China; ^3^Institute of Grassland Research, Chinese Academy of Agricultural Sciences, Hohhot, China

**Keywords:** grazing management, greenhouse gas, alpine meadow, methane flux, methane sink, methane-oxidizing bacteria

## Abstract

Grazing exclusion (GE) is an effective measure for restoring degraded grassland ecosystems. However, the effect of GE on methane (CH_4_) uptake and production remains unclear in dominant bacterial taxa, main metabolic pathways, and drivers of these pathways. This study aimed to determine CH_4_ flux in alpine meadow soil using the chamber method. The *in situ* composition of soil aerobic CH_4_-oxidizing bacteria (MOB) and CH_4_-producing archaea (MPA) as well as the relative abundance of their functional genes were analyzed in grazed and nongrazed (6 years) alpine meadows using metagenomic methods. The results revealed that CH_4_ fluxes in grazed and nongrazed plots were −34.10 and −22.82 μg‧m^−2^‧h^−1^, respectively. Overall, 23 and 10 species of Types I and II MOB were identified, respectively. Type II MOB comprised the dominant bacteria involved in CH_4_ uptake, with *Methylocystis* constituting the dominant taxa. With regard to MPA, 12 species were identified in grazed meadows and 3 in nongrazed meadows, with *Methanobrevibacter* constituting the dominant taxa. GE decreased the diversity of MPA but increased the relative abundance of dominated species *Methanobrevibacter millerae* from 1.47 to 4.69%. The proportions of type I MOB, type II MOB, and MPA that were considerably affected by vegetation and soil factors were 68.42, 21.05, and 10.53%, respectively. Furthermore, the structural equation models revealed that soil factors (available phosphorus, bulk density, and moisture) significantly affected CH_4_ flux more than vegetation factors (grass species number, grass aboveground biomass, grass root biomass, and litter biomass). CH_4_ flux was mainly regulated by serine and acetate pathways. The serine pathway was driven by soil factors (0.84, *p* < 0.001), whereas the acetate pathway was mainly driven by vegetation (−0.39, *p* < 0.05) and soil factors (0.25, *p* < 0.05). In conclusion, our findings revealed that alpine meadow soil is a CH_4_ sink. However, GE reduces the CH_4_ sink potential by altering vegetation structure and soil properties, especially soil physical properties.

## 1 Introduction

Methane (CH_4_) is considered the second most important greenhouse gas following carbon dioxide (CO_2_). However, the potential of CH_4_ to affect global warming is ~28 times that of CO_2_ (Stocker et al., 2013). Furthermore, CH_4_ is responsible for ~20% of global warming effects, thus profoundly affecting the rate of global warming. In the global CH_4_ budget, terrestrial soil constitutes the most substantial biological sink of atmospheric CH_4_ (Dutaur and Verchot, 2007; De Bernardi et al., 2022). Grassland soil absorbs 3.73 Tg of CH_4_ annually (Yu et al., 2017), and alpine meadows on the Qinghai–Tibet Plateau exhibit the highest CH_4_ uptake (0.284 Tg of CH_4_ annually), accounting for ~44% of the total CH_4_ absorption by grassland soil in China (Wang et al., 2014). Therefore, CH_4_ metabolism in alpine meadows is critical for its effects on global climate change.

Methanotrophs catalyze CH_4_ oxidation, they are classified as aerobic CH_4_-oxidizing bacteria (MOB) and anaerobic methanotrophic archaea (ANME), which can offset the atmospheric effects of CH_4_ emissions (Knief, 2015; Schmitz et al., 2021; Wallenius et al., 2021). MOB can be categorized into the following four groups according to their physiological, phylogenetic, biochemical, and phenotypical characteristics: type I MOB (Gammaproteobacteria), type II MOB (Alphaproteobacteria), Verrucomicrobia, and NC10 (Jeremy et al., 2010; Knief, 2015; Padilla et al., 2016). Alphaproteobacteria and Gammaproteobacteria are abundant in CH_4_-metabolizing flora and have been studied for more than a century (Schmieder and Edwards, 2011). Types I and II MOB assimilate CH_4_ via the ribulose monophosphate pathway (RuMP) and serine pathway, respectively (Hanson and Hanson, 1996; Jeremy et al., 2010). ANME belong to the Euryarchaeota phylum and include ANME-1, ANME-2, and ANME-3. They are mainly found in anoxic environments such as coastal sediments, wetland soils, and water bodies (Wallenius et al., 2021). Methanogens, belonging to archaea, are CH_4_-producing microorganisms; therefore, they are also known as CH_4_-producing archaea (MPA). In terms of taxonomy, MPA was found within 8 orders, including Methanococcales, Methanopyrales, Methanobacteriales, Methanomicrobiales, Methanocellales, Methanonatronarchaeales, Methanosarcinales, and Methanomassiliicoccales (Lyu et al., 2018). Further, the following three methanogenic pathways have been reported (KEGG, 2022): CO_2_ to CH_4_ (CO_2_ pathway), methanol to CH_4_ (methanol pathway), and acetate to CH_4_ (acetate pathway). Analysis of functional genes is an effective means of explaining the taxonomic and functional changes in microbial communities. Recent studies have evaluated the CH_4_ uptake and production potential of microbial communities by detecting monooxygenase subunit A (*pmoA*) and methyl coenzyme M reductase A (*mcrA*) genes, respectively (Bhardwaj and Dubey, 2020; Zhang et al., 2022). Although analysis of *pmoA* and *mcrA* effectively indicates alterations in the abundance of MOB and MAP, such analysis cannot determine the differences between methanotrophic and methanogenic pathways. Recent evidence suggests that gene abundance determined using metagenomics can predict CH_4_ metabolism more effectively than community profiling (Zhang et al., 2022).

Overgrazing can convert an atmospheric CH_4_ sink to a CH_4_ source in the rangeland ecosystem, and CH_4_ emission is known to increase with increasing grazing intensity (Tang et al., 2019). This process can be reversed via grazing exclusion (GE), which alters the plant community structure and soil properties (Liu et al., 2015; Li et al., 2019; Dang et al., 2023). Vegetation and soil factors influence the composition and function of the soil microbiome (Upton et al., 2020; Wang et al., 2022). The physical properties of soil are vital for CH_4_ circulation. Priano et al. (2018) reported that CH_4_ metabolism is primarily related to soil texture and that CH_4_ diffusivity in soil is affected by differences in silt and clay contents and soil bulk density, which in turn affect CH_4_ emissions. A previous study showed that CH_4_ uptake is positively correlated with soil moisture, temperature, and precipitation (Yue et al., 2016). Additionally, livestock is an essential driver of grassland vegetation succession, and increased quantity of leguminous forage promotes CH_4_ emission (Khalsa et al., 2014). Thus, plant community structure and soil physicochemical properties regulate methanotrophic and methanogenic communities.

In the past decades, owing to heavy grazing, alpine meadows on the Qinghai–Tibet Plateau have been largely degraded. In 2014, we conducted a GE experiment to track the restoration of the ecosystem. Currently, vegetation biomass and soil fertility could be considerably restored after fencing (Li et al., 2018). However, the underlying mechanism of CH_4_ metabolism in the plant growing season remains unclear. In the present study, *in situ* CH_4_ flux was determined in August 2020, and metagenomics was employed to analyze the composition of methanotrophic and methanogenic communities as well as the relative abundance of their functional genes in the shallow soil of grazed and nongrazed (2014–2020) alpine meadows. A 6-year study is necessary because the stability of the vegetation community and soil properties in alpine meadows reaches an optimum level only after 6 years of GE (Li et al., 2018). The resultant metagenomic, vegetation, and soil data were combined to (1) determine the effects of GE on the dominant CH_4_-metabolizing taxa in degraded alpine meadows; (2) identify the main CH_4_ uptake and production pathways based on alterations in related functional genes; (3) explore the heterogeneous responses of methanotrophic and methanogenic communities to GE. By analyzing the response of CH_4_ metabolic communities and functional genes to grazing exclusion, we will elucidate the key environmental factors regulating CH_4_ bioprocesses and provide a theoretical guide for decision making in natural restoration of grasslands by fencing.

## 2 Materials and methods

### 2.1 Site description and experimental design

**Figure 1:**
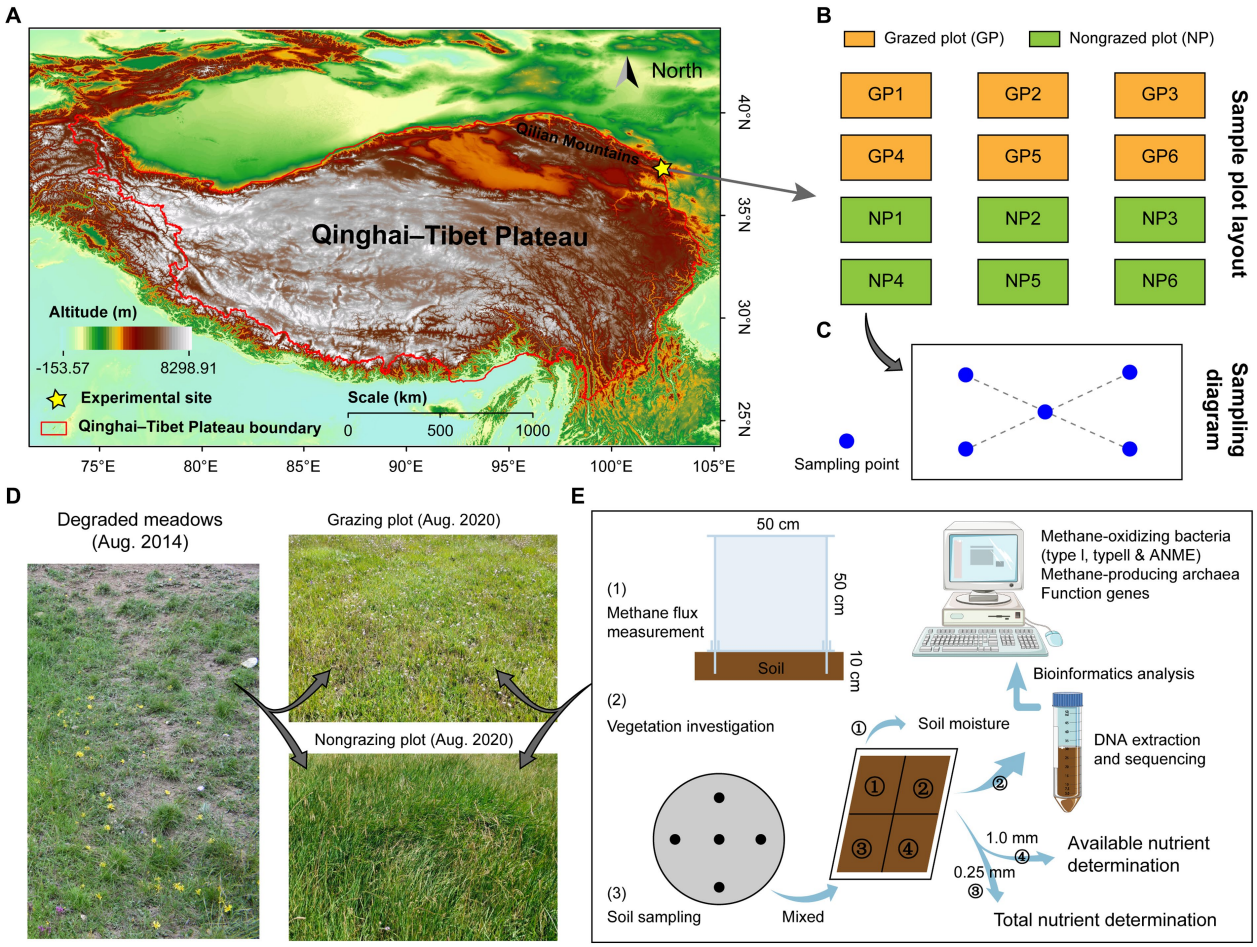
Experimental design and steps. Grazed and nongrazed plots were established in alpine meadows on the eastern Qinghai-Tibet Plateau in May 2014 **(A)**, with six replicates in grazed and nongrazed plots **(B)**. **(C)** Shows the sampling diagram of vegetation survey and soil sampling in August 2020, and blue dots indicate sampling points. **(D)** Shows the vegetation landscape of grazed and nongrazed plots. **(E)** Shows the experimental steps, soil was collected at a depth of 0–10 cm at each sampling point (five collections) and mixed into one sample. The pooled soil samples were divided into four parts using the tetrad method: One part was used to measure moisture, one part was cryopreserved (−80°C) for DNA extraction and sequencing, one part was used to determine total nutrient content, and one part was used to determine available nutrient content. Further details are provided in the main text.

The study site was located at the northeastern edge of the Qinghai–Tibetan Plateau in Tianzhu Tibetan Autonomous County, Gansu Province, China (37°10′12.92″N, 102°47′12.12″E; 3042.4 m above sea level, Figure 1A). This site exhibits a considerable temperature difference between day and night, has a cold and wet climate, and has no absolute frost-free period (Wang et al., 2020). The annual rainfall is 467.9 mm, with 76% of the rainfall being concentrated from June to September. The average annual temperature is 1.1°C, with the average temperature in July (the warmest month) being 13.7°C and that in January (the coldest month) being −10.3°C (Wang et al., 2022). Plants grow for ~120 days throughout the year, and alpine meadows comprise the primary vegetation type at the study site. The dominant herbages are mainly from the Gramineae, Salicaceae, and Polygonaceae families, and the dominant species include *Poa pratensis*, *Elymus nutans*, *Polygonum viviparum*, *Kobresia humilis*, *Blysmus sinocompressus*, and *Kobresia capillifolia*. Based on vegetation and soil characteristics before 2014, the meadowland at the experimental site was heavily degraded, according to a standard for alpine meadow degradation by Ma et al. (2002). A region with uniform vegetation distribution in the alpine meadows was selected as the experimental site in May 2014. Grazed plots (GPs) and nongrazed plots (NPs) were established in the degraded alpine meadows. Six replicates were used per plot, each with an area of 100 m × 50 m (Figure 1B). The grazing intensity in GPs was four sheep/ha, and grazing occurred continuously throughout the year. All types of grazing were prohibited in NPs from 2014 to 2020.

### 2.2 CH_4_ flux, vegetation, and soil data collection

In August 2020, acrylic chambers were used to determine CH_4_ flux *in situ* in GPs and NPs. An acrylic chamber consists of two parts: a base (50 cm × 50 cm × 15 cm) inlaid into 10-cm deep soil and a cube box (50 × 50 × 50 cm^3^) covering the base. The connection between the base and the box was sealed with water. In the plots, 12 chambers were installed and vegetation was cut. CH_4_ flux was measured at 9:00–11:00 daily for 1 week, and the gas in the box was collected using a syringe every 15 min (Xing et al., 2022). The box was covered with a white insulating foam during sampling to avoid the warming effect of sunlight (Zhang et al., 2013). Subsequently, the gas samples were analyzed within 48 h using a gas chromatograph (Agilent 6820A, CA, United States). CH_4_ was tested using a flame ionization detector, and N_2_ was used as the carrier gas to remove O_2_ and water vapor (Chen et al., 2017). The CH_4_ flux was calculated according to the method reported by Xing et al. (2022).

Vegetation and soil indices were measured in GPs and NPs (Figure 1D). To reduce the differences caused by soil spatial heterogeneity, 5 sampling points were selected in each plot using the “X” sampling method and mixed into one sample (Figure 1C), thus obtaining a total of 12 samples. To avoid boundary effects, the distance between all sampling points and the fence was maintained at >5 m. Soil temperature (ST) was measured at a depth of 0–10 cm at each sampling site for a week under clear weather conditions. Vegetation and litter features were surveyed using a sample square (1 m^2^) at each sampling site. In the sampling square, grass species, individual numbers, plant height (using a tape measure), and coverage were recorded. The aboveground biomass (including mosses) was determined via mowing, and the litter was collected. Furthermore, a soil auger (inner diameter, 3.5 cm) was used to collect soil at a depth of 0–10 cm. From the same sampling point, five samples were collected and pooled into one sample. The quadrat method was used to divide the soil into four parts (Figure 1E). The soil indicators included soil organic matter (SOM), available nitrogen (AN), available phosphorus (AP), available potassium (AK), total nitrogen, total phosphorus (TP), and total potassium (TK), which were measured according to the methods described by Bao (2000). The soil samples were stored at −80°C for DNA extraction and sequencing (six repetitions). Additionally, soil was collected from the sample squares at a depth of 0–10 cm using a soil auger (diameter, 10 cm), packed in mesh bags, and rinsed with water to obtain the plant roots. Soil samples were also collected from the sample squares using a cutting ring (diameter, 5 cm; height, 5 cm) and dried to a constant weight at 105°C, following which soil bulk density (SBD) and soil moisture (SM) were determined. All aboveground plant, root, and litter samples were dried to a constant weight at 70°C and then weighed to calculate grass aboveground biomass (GAB), grass root biomass (GRB), and litter biomass (LB). The importance value of the grass species was calculated as follows (Ren et al., 1998):


$$\text{Importance value}=\frac{H^{\prime }+C^{\prime }+D^{\prime }+B^{\prime }}{4}$$

where $H^{\prime }$, $C^{\prime }$, $D^{\prime }$, and $B^{\prime }$ indicate the relative values of grass height, coverage, density, and GAB, respectively.

### 2.3 DNA extraction, sequencing, and bioinformatics analysis

Genomic DNA was extracted from the soil samples using hexadecyltrimethyl ammonium bromide (Mackelprang et al., 2011; Hultman et al., 2015). DNA degradation and potential contamination were detected via 1% agarose gel electrophoresis. DNA concentration was accurately measured using Qubit dsDNA Assay Kit and Qubit 2.0 Fluorescence Quantification Instrument (Life Technologies, CA, United States). The tested DNA was randomly spliced to obtain ~350-bp fragments using Covaris ultrasonic fragmentation device. End repair, A-tail addition, sequencing connector addition, purification, and polymerase chain reaction (PCR) amplification were performed. Libraries were constructed using NEB Next Ultra DNA Library Prep Kit for Illumina (NEB, MA, United States). The PCR products were purified and the library size distribution was analyzed using Agilent 2100 Bioanalyzer. Indexed coded samples were clustered using cBot Cluster Generation System according to the manufacturer’s instructions. Library preparations were sequenced using the Illumina NovaSeq6000 platform, generating pair-end sequences.

Metagenome sequencing was performed using the Illumina NovaSeq high-throughput sequencing platform to obtain data regarding raw bacteria, fungi, and archaea from 0 to 10-cm deep soil samples. The raw sequences were preprocessed to ensure the reliability of the data. In particular, the sequencing junction sequences were removed from the raw data using cutadapt, and quality control of the data was performed using KneadData software. FastQC was used to assess the rationality and effectiveness of the quality control process (Schmieder and Edwards, 2011). Following quality control, the processed data were compared with the host genome data using Bowtie2, and sequences that were not comparable to those of the host were retained for subsequent analysis. The quality of the sequencing data is shown in Supplementary Table S1. Kraken2 and a self-constructed microbial database were used for species annotation to identify the species present in the samples, and Bracken was used to predict their relative abundance (Lu et al., 2017). Kraken2 is the latest comparison software based on K-mer, and the local Kraken2 database contained 16,799 known bacterial genomes. Finally, functional annotation was performed based on reads (Wood and Salzberg, 2014) using HUMAnN2 software to match the reads of the individual samples with those in the database (UniRef90). Based on this analysis, annotation information and relative abundance tables were obtained for each functional database. Microbial biomarkers were identified at the species level using the linear discriminant analysis (LDA) effect size (LEfSe) method based on an LDA score of >2. Additionally, reads per kilobase per million mapped read values were used to estimate the relative abundance of functional genes (Kim et al., 2016). Default parameters were used for the abovementioned analyses.

### 2.4 Statistical analysis

Data were tested for normality and homogeneity of variances. Independent samples *t*-test was conducted between the two groups (GPs and NPs) when the data followed a normal distribution in Shapiro–Wilk and Levene’s tests. The data are presented as means ± standard errors, and statistical significance was indicated by a *p*-value of <0.05. If the data followed a non-normal distribution in Shapiro–Wilk or Levene’s test, a nonparametric test (Mann–Whitney U test) was used. Additionally, redundancy analysis was performed to determine the potential associations of drivers with CH_4_-metabolizing microbial communities and their functions. The random forest method was used to select soil indicators and construct soil composite variables. Structural equation models (SEMs) were used to analyze the interactions among vegetation, soil, microbial taxa, metabolic pathways, and CH_4_ flux. Furthermore, Spearman’s correlation analysis and environmental factor mapping with microbial composition and function were performed. The abovementioned analysis was performed using R version 4.2.2 with the R packages randomForest, Pheatmap, corrplot, piecewiseSEM, and ggplot2. Finally, a microbial co-occurrence network diagram (ρ > 0.7; *p* < 0.05) was constructed and mapped using BioinCloud.^1^

## 3 Results

### 3.1 Variability in CH_4_ flux and environmental properties

**Figure 2:**
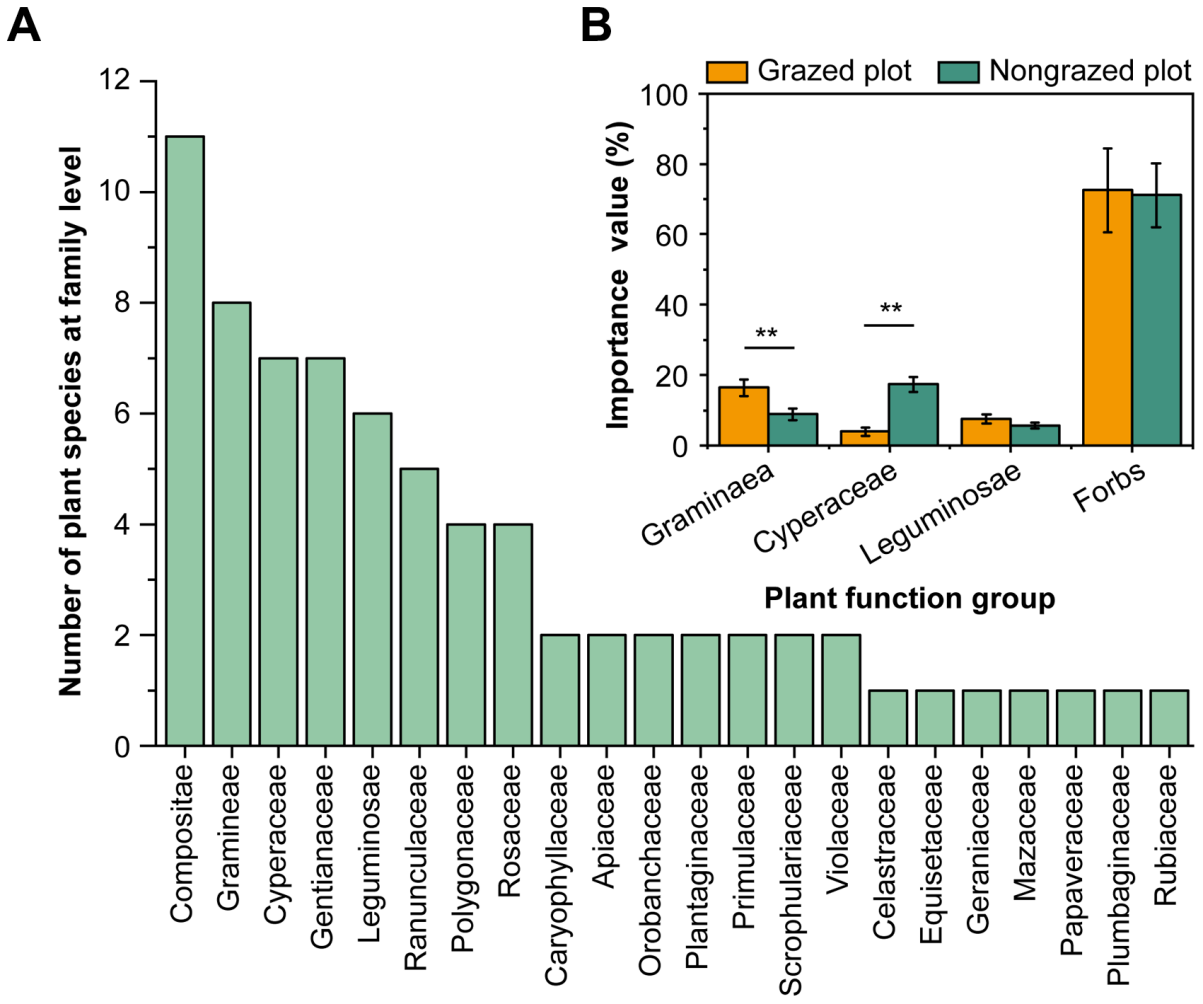
Plant community composition at the family level and its importance values at the experimental site. **(A)** Grass species number at the family level. **(B)** Response of the importance values of the plant functional group to GE. **p < 0.01.

GE altered the CH_4_ flux, vegetation characteristics, and soil properties of the study site (Table 1). Although alpine meadow soil is a CH_4_ sink, compared with grazing, GE increased the CH_4_ flux of the 0–10-cm deep soil from −34.10 to −22.82 μg‧m^−2^‧h^−1^. In total, 73 grass species belonging to 46 genera and 22 families were recorded in the experimental plots (Figure 2A; Supplementary Table S2). The top five families in terms of species richness were Compositae, Gramineae, Cyperaceae, Gentianaceae, and Leguminosae. All species were categorized into four functional groups (Gramineae, Cyperaceae, Leguminosae, and forbs). GE decreased the importance value of Gramineae by 46.31% and increased the importance value of Cyperaceae by 388.03% (Figure 2B). Notably, a high proportion of forbs, particularly *Bistorta vivipara*, *Equisetum arvense*, and *Thalictrum alpinum*, was detected at the experimental site (Supplementary Table S2). GAB and GRB were higher in NPs than in GPs (*p* < 0.01), and grass species number (GSN) decreased by 40.30% after fencing. LB was 2.34 times higher in NPs than in GPs. Furthermore, compared with GPs, soil aggregates (SAs; >1.0 mm) and SM increased in NPs by 17.12 and 49.62%, respectively. However, SBD and pH were lower in NPs than in GPs. Regarding soil nutrition, the SOM, AN, AP, AK, TP, and TK contents detected in the soil were higher after GE than before GE.

**Table 1:** Effects of GE on CH_4_ flux and environmental features.

Environment factors	Grazed plot (GPs)	Nongrazed plot (NPs)
CH_4_ flux (μg‧m^−2^‧h^−1^)	−34.10 ± 1.99	−22.82 ± 2.70^**^
GSN	22.33 ± 1.25	13.33 ± 1.16^**^
GAB (g/m^2^)	86.85 ± 3.01	504.58 ± 10.53^***^
GRB (g/m^2^)	605.55 ± 29.14	840.37 ± 54.79^**^
LB (g/m^2^)	17.48 ± 2.23	58.43 ± 2.35^***^
SBD (g/m^3^)	1.08 ± 0.04	0.84 ± 0.01^***^
SAs (>1.0 mm, %)	55.96 ± 2.1	65.54 ± 1.69^**^
ST (°C)	10.58 ± 0.25	9.68 ± 0.19^*^
SM (%)	25.53 ± 0.25	38.20 ± 0.54^***^
pH	7.04 ± 0.01	7.03 ± 0.01
SOM (g/kg)	169.15 ± 2.65	191.52 ± 6.88^*^
AN (mg/kg)	470.50 ± 12.04	528.40 ± 14.29^*^
AP (mg/kg)	7.25 ± 0.5	8.50 ± 0.18^*^
AK (mg/kg)	501.20 ± 7.55	533.77 ± 7.10^**^
TN (g/kg)	10.38 ± 1.03	8.64 ± 0.38
TP (g/kg)	0.73 ± 0.03	0.80 ± 0.02
TK (g/kg)	7.10 ± 0.12	7.45 ± 0.14

GSN, grass species number; GAB, grass aboveground biomass; GRB, grass root biomass; LB, litter biomass; SBD, soil bulk density; SAs, soil aggregates; ST, soil temperature; SM, soil moisture; SOM, soil organic matter; AN, available nitrogen; AP, available phosphorus; AK, available potassium; TN, total nitrogen; TP, total phosphorus; TK, total potassium.*p < 0.05, **p < 0.01, ***p < 0.001.

### 3.2 Community composition of MOB and MPA

**Figure 3:**
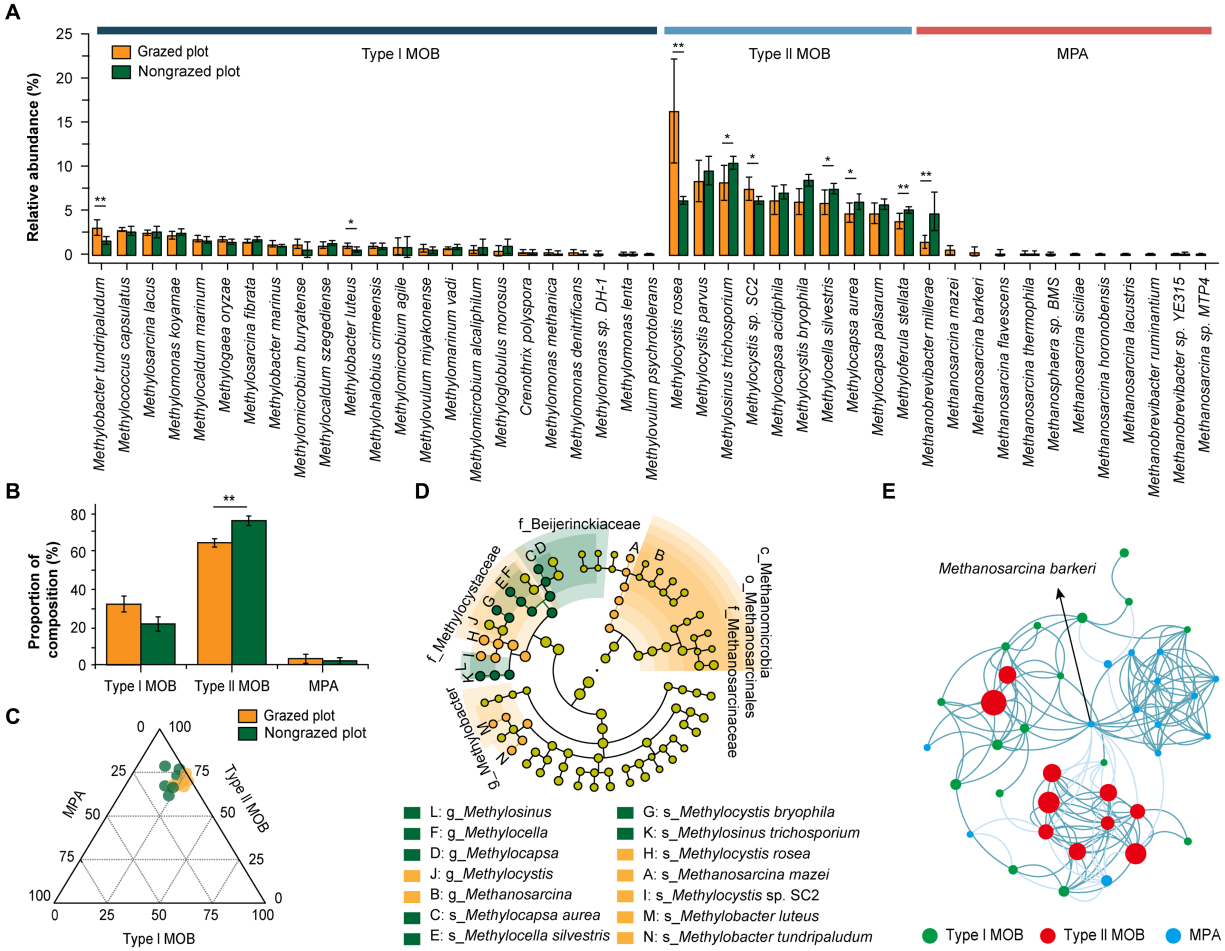
Response of microbial communities associated with CH_4_ metabolism to GE. **(A)** Relative abundance of type I MOB, type II MOB, and MPA at the species level (six repetitions). **(B)** Effect of GE on the relative abundance of type I MOB, type II MOB, and MPA. **(C)** A ternary diagram illustrating the compositions of type I MOB, type II MOB, and MPA. The size of the bubbles indicates the sum of the relative abundance of type I MOB, type II MOB, and MPA taxa. **(D)** Microbial biomarkers for CH_4_ metabolism at different taxonomic levels according to the linear discriminant analysis (LDA) effect size method (LDA score of >2). Uppercase letters indicate biomarkers, whereas lowercase letters indicate microbial taxa at different levels: c, class; o, order; f, family; g, genus; s, species. **(E)** Co-occurrence networks of soil microbial species. Visualization of the connectivity in type I MOB, type II MOB, and MPA taxa. Nodes indicate individual microbial species, and edges represent significant Spearman correlations (ρ > 0.7; *p* < 0.05). The size of the nodes indicates the relative abundance of species. Dark cyan edges indicate positive correlations, and light cyan edges indicate negative correlations. *p < 0.05, **p < 0.01.

Table 2 presents the composition of MOB and MPA at family and genus levels as well as their relative abundances in response to GE. ANME was not detected in the soil samples. The relative abundance of MOB accounted for 0.34 and 0.30% of the total microorganisms in GPs and NPs, respectively. And the relative abundance of MPA accounted for 0.012 and 0.015% of the total microorganisms in GPs and NPs, respectively. Regarding CH_4_ uptake, type I MOB comprised 23 species from 12 genera and type II MOB comprised 10 species from 4 genera (Table 2 and Figure 3A). Conversely, Verrucomicrobia or the NC10 phylum was not detected in the analyzed samples. The level of species diversity in type I MOB was higher than that in type II MOB. However, their relative abundances exhibited the opposite trend. The relative abundances of type I MOB were 32.14% in GPs and 21.59% in NPs and those of type II MOB were 64.64% in GPs and 76.34% in NPs. Furthermore, GE decreased the relative abundances of type II MOB (Figure 3B). Among all MOB, the top five genera in terms of relative abundance were *Methylocystis*, *Methylocapsa*, *Methylosinus*, *Methylocella*, and *Methylobacter*, four of which belonged to type II MOB (Table 2). The top five species in terms of relative abundance were *Methylocystis rosea*, *Methylosinus trichosporium*, *Methylocystis parvus*, *Methylocystis bryophila*, and *Methylocystis* sp. SC2, all of which belonged to type II MOB (Figure 3A). These results revealed that type II MOB was the dominant taxa associated with CH_4_ uptake, indicating that the serine pathway plays a dominant role in CH_4_ uptake. Regarding CH_4_ production, MPA exhibited lower diversity and relative abundance than MOB. MPA comprised 3 genera (*Methanobrevibacter*, *Methanosarcina*, and *Methanosphaera*) and 12 species (Table 2, Figure 3A). *Methanobrevibacter* and *Methanosarcina* constituted the dominant genera, whereas *Methanobrevibacter millerae* was the absolute dominant species. GE increased the relative abundance of *Methanobrevibacter*, and *Methanosarcina* was not found in NPs. Additionally, GE reduced the relative abundance of MPA species by 35.71%, leading to the disappearance of nine species. However, the relative abundance of *M. millerae* increased significantly from 1.47 to 4.69% (*p* < 0.01).

**Table 2:** CH_4_-metabolizing microbial taxa and their relative abundances at the family and genus levels.

Family	Genus	Relative abundance (%)	
		Grazed plots (GPs)	Nongrazed plots (NPs)
**Type I methane-oxidizing bacteria**			
Methylococcaceae	*Methylobacter*	5.35 ± 0.52	3.23 ± 0.28*
Methylococcaceae	*Methylosarcina*	3.99 ± 0.24	4.28 ± 0.39
Methylococcaceae	*Methylomonas*	2.99 ± 0.27	3.02 ± 0.35
Methylococcaceae	*Methylomicrobium*	2.63 ± 0.66	2.15 ± 1.01
Methylococcaceae	*Methylocaldum*	2.87 ± 0.33	2.88 ± 0.30
Methylococcaceae	*Methylococcus*	2.79 ± 0.23	2.66 ± 0.49*
Methylococcaceae	*Methylohalobius*	0.99 ± 0.32	0.94 ± 0.40
Methylococcaceae	*Methylogaea*	1.76 ± 0.30	1.46 ± 0.32
Methylococcaceae	*Methyloglobulus*	0.39 ± 0.57	0.96 ± 0.77
Methylococcaceae	*Methylomarinum*	0.71 ± 0.19	0.90 ± 0.32
Methylococcaceae	*Methylovulum*	0.77 ± 0.24	0.50 ± 0.29
Crenotrichaceae	*Crenothrix*	0.26 ± 0.28	0.17 ± 0.24
**Type II methane-oxidizing bacteria**			
Beijerinckiaceae	*Methylocapsa*	15.60 ± 1.08	18.81 ± 0.68*
Beijerinckiaceae	*Methylocella*	5.91 ± 1.45	7.46 ± 0.58
Beijerinckiaceae	*Methyloferula*	3.80 ± 0.83	5.05 ± 0.43
Methylocystaceae	*Methylocystis*	38.10 ± 3.17	30.25 ± 0.61**
Methylocystaceae	*Methylosinus*	8.13 ± 1.94	10.38 ± 0.79*
**Methane-producing archaea**			
Methanobacteriaceae	*Methanobrevibacter*	1.82 ± 0.11	1.92 ± 2.13*
Methanobacteriaceae	*Methanosphaera*	0.15 ± 0.21	0.15 ± 0.20
Methanosarcinaceae	*Methanosarcina*	1.25 ± 0.26	–

*p < 0.05, **p < 0.01.

As shown in the ternary diagram in Figure 3C, type II MOB was the most abundant among all CH_4_-metabolizing microbial communities. The LEfSe results revealed the presence of 12 and 8 biomarkers in GPs and NPs, respectively (Figure 3D). Methanmicrobia, Methanosarcinales, Methanosarcinaceae, and Methylocystaceae were the biomarkers in GPs, whereas Beijerinckiaceae was the biomarker in NPs. At the species level, *Methylocapsa aurea*, *Methylocella silvestris*, *M. bryophila*, and *M. trichosporium* were the biomarkers in NPs, whereas *M. rosea*, *Methanosarcina mazei*, *M.* sp. SC2, *Methylobacter luteus*, and *Methylobacter tundripaludum* were the biomarkers in GPs. The positive correlation among the species exceeded the negative correlation, and the top five species in terms of positive correlation were *Methanosarcina barkeri*, *M. aurea*, *Methylocapsa palsarum*, *M. trichosporium*, and *M. silvestris*. Among all species, *M. barkeri* was at the center of the network, with a maximum value of 28.75 for connectivity (Figure 3E).

### 3.3 Methanotrophic and methanogenic functional genes

**Figure 4:**
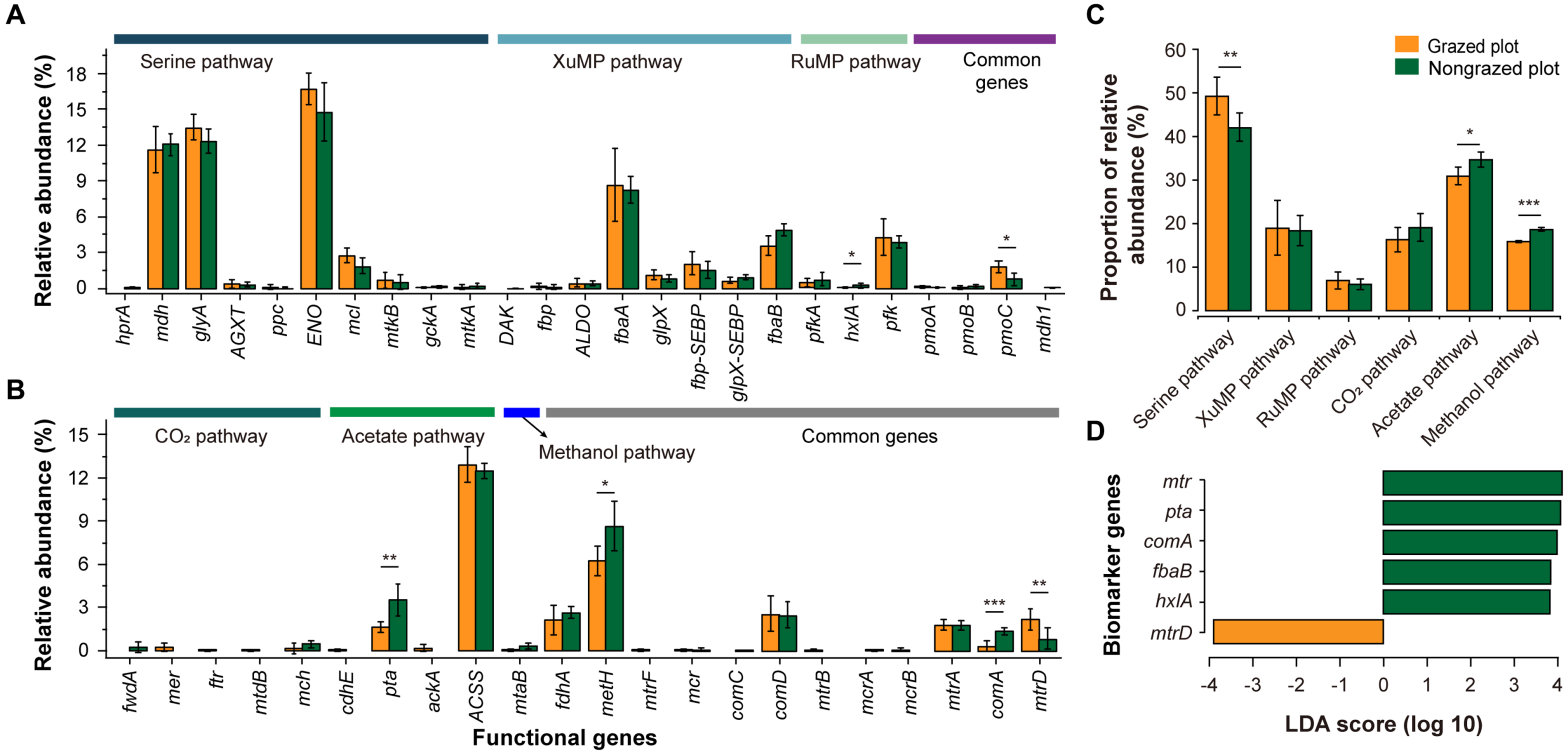
Response of the relative abundance of functional genes related to CH_4_ uptake **(A)** and production **(B)** to GE in GPs and NPs (six repetitions). **(C)** Relative abundance of genes involved in the different pathways of CH_4_ uptake and production. **(D)** Biomarker genes for CH_4_ metabolism according to the linear discriminant analysis effect size method (LDA score of >2). *p < 0.05, **p < 0.01, ***p < 0.001.

In total, 25 and 22 functional genes associated with methanotrophic and methanogenic processes, respectively, were analyzed (Figure 4; Supplementary Figure S1). Among CH_4_ uptake-related genes, the top five functional genes in terms of relative abundance were *ENO*, *glyA*, *mdh*, *fbaA*, and *pfk*. Among them, three functional genes (*ENO*, *glyA*, and *mdh*) belonged to the serine pathway. The relative abundance of functional genes related to the serine pathway was higher than that of functional genes related to xylulose monophosphate (XuMP) and RuMP pathways (Figure 4C). Furthermore, GE decreased significantly the relative abundance of functional genes related to the serine pathway (*p* < 0.01). Among CH_4_ production-related genes, the top five functional genes in terms of relative abundance were *ACSS*, *metH*, *pta*, *comD*, and *fdhA*. The relative abundances of *ACSS* and *metH* were higher than those of other functional genes related to the CH_4_ production pathway. The order of the three pathways in terms of relative abundance was acetate pathway > CO_2_ pathway > methanol pathway. GPs exhibited a lower relative abundance of functional genes related to the CH_4_ production pathway than NPs (Figure 4C). One biomarker gene (*mtrD*) was detected in GPs and five biomarker genes (*metH*, *pta*, *comA*, *fbaA*, and *hxlA*) were detected in NPs (Figure 4D).

### 3.4 Associations with vegetation and soil factors

**Figure 5:**
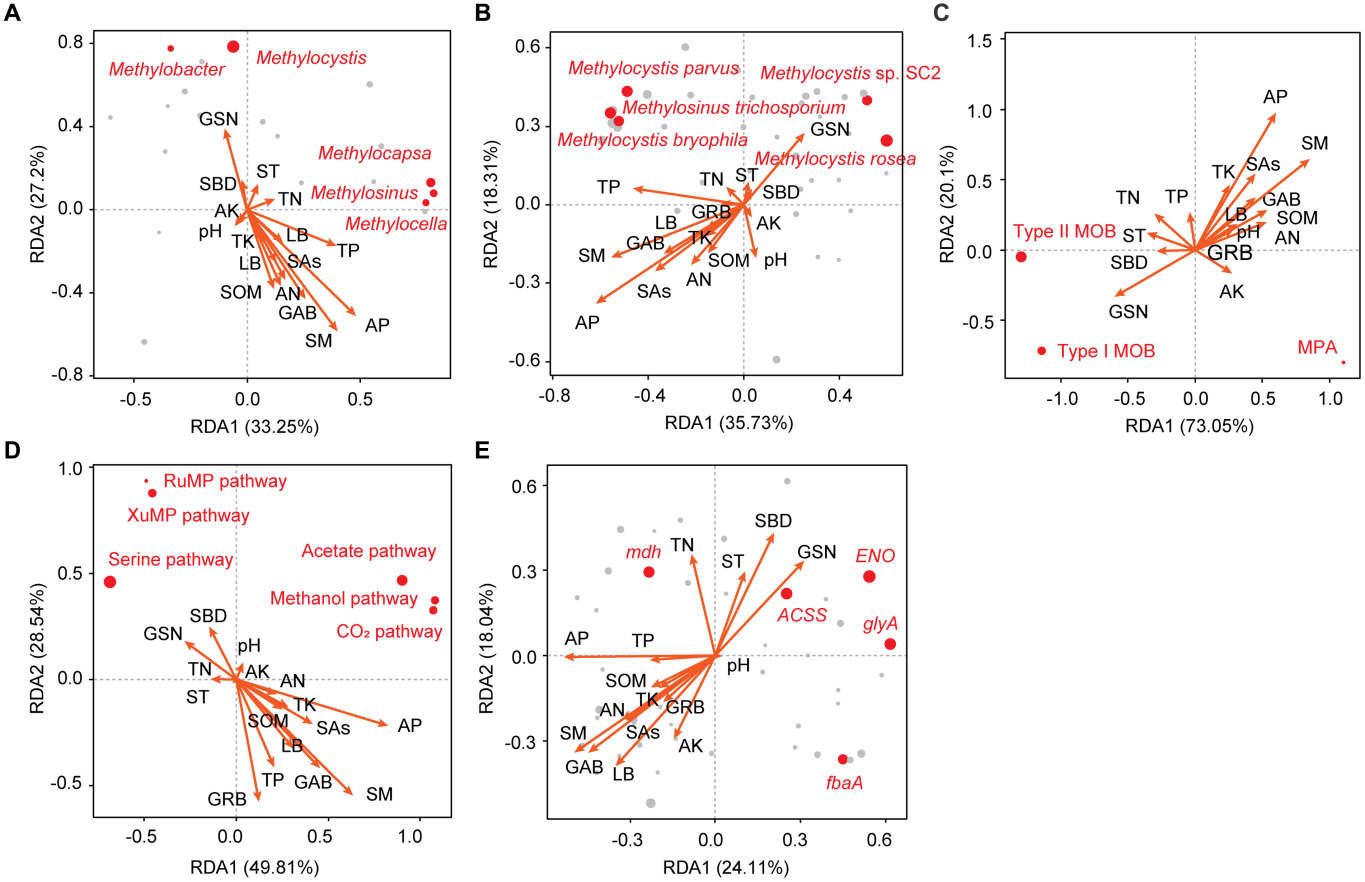
Redundancy analysis of vegetation features, soil properties, and CH_4_ metabolism-related taxa at the genus **(A)** and species **(B)** levels; type I MOB, type II MOB, and MPA **(C)**; CH_4_ uptake and production pathways **(D)**; and functional genes **(E)**. Red dots represent the top five genera in **(A)**, species in **(B)**, and functional genes in **(D)** in terms of relative abundance. The size of the points reflects the relative abundance. GSN, grass species number; GAB, grass aboveground biomass; GRB, grass root biomass; LB, litter biomass; SBD, soil bulk density; SAs, soil aggregates; ST, soil temperature; SM, soil moisture; SOM, soil organic matter; AN, available nitrogen; AP, available phosphorus; AK, available potassium; TN, total nitrogen; TP, total phosphorus; TK, total potassium. The same as below.

**Figure 6:**
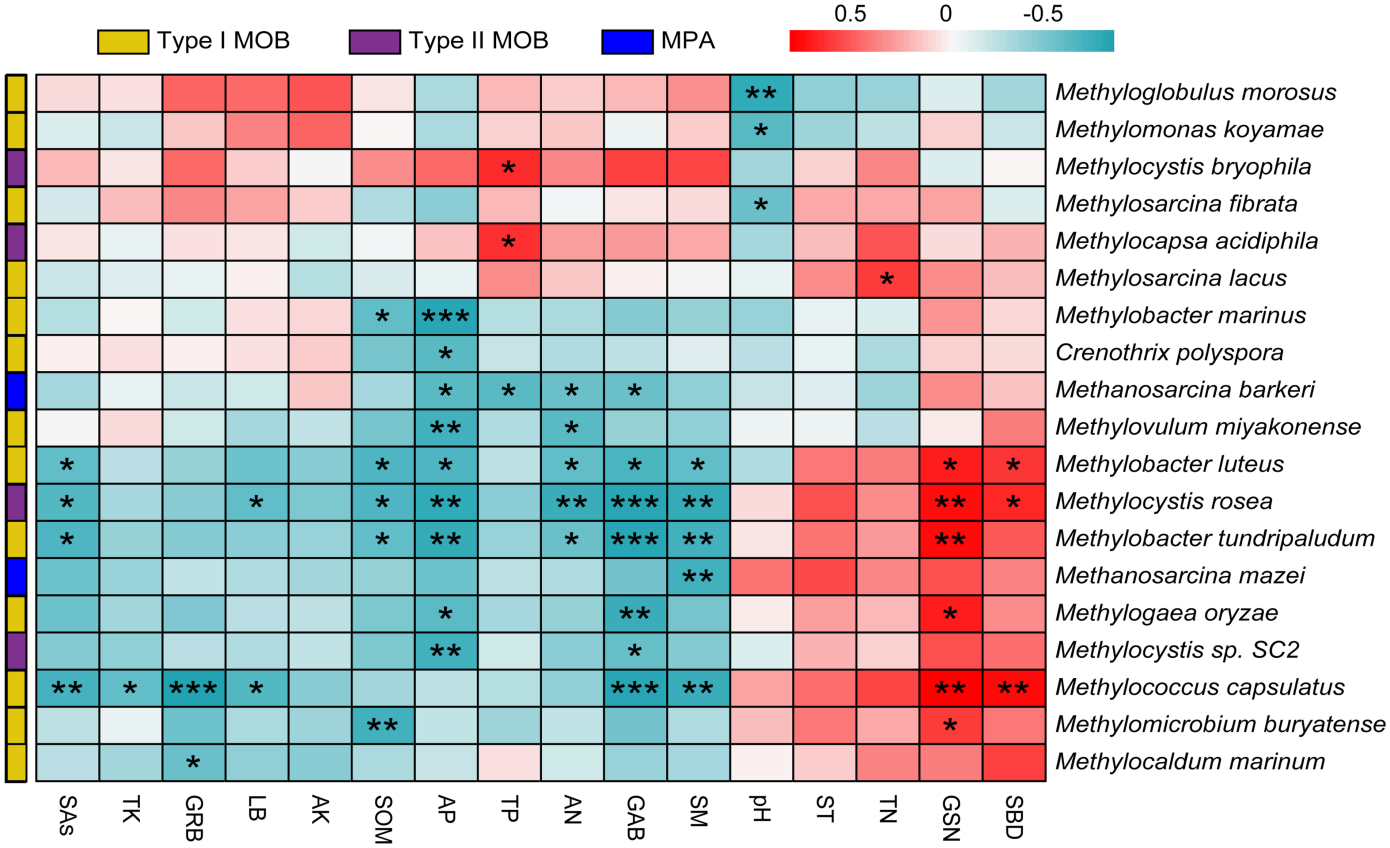
Correlation analysis including MOB and MPA at the species level and environmental factors. Red indicates a positive relationship and blue indicates a negative relationship. Features with no significant correlation were removed. *p < 0.05, **p < 0.01, ***p < 0.001.

The responses of MOB and MPA communities and functional genes to vegetation and soil factors were analyzed. The top five influential factors were SM, AP, GSN, GAB, and TP for microbial taxa at the genus level (Figure 5A) and AP, SM, GSN, TP, and SAs for microbial taxa at the species level (Figure 5B). Overall, 10 genera were significantly affected by vegetation and soil factors (*p* < 0.05); 8 of these genera were type I MOB, 1 genus was a type II MOB, and 1 genus was a type MPA (Supplementary Figure S2). Overall, 19 species were significantly affected by vegetation and soil factors, and 68.42, 21.05, and 10.53% of them were type I MOB, type II MOB, and MPA, respectively (Figure 6). Thus, type I MOB were more likely to be influenced by vegetation and soil factors than type II MOB and MPA. The influence of environmental factors varied in different species. Among the top five genera in terms of relative abundance, *Methylobacter* and *Methylocistis* were primarily negatively correlated with environmental factors. Conversely, *Methylocapsa*, *Methylosinus*, and *Methylocella* were primarily positively correlated with environmental factors. *M. parvus* and *M. bryophila*, exhibiting the highest relative abundance, were not significantly correlated with the 16 environmental factors and showed strong stability. Conversely, *M. rosea* was significantly positively correlated with GSN and SBD and significantly negatively correlated with SAs, LB, SOM, AP, AN, SM, and GAB. The trend observed for *M. tundripaludum* and *Methylococcus capsulatus* was similar to that observed for *M. rosea*, i.e., they were insensitive to the effects of environmental factors (Figures 5B, 6). Type I MOB were negatively correlated with 4 environmental factors and positively correlated with 12 environmental factors, with 72.31% of the correlations being significant. Type II MOB were negatively correlated with 5 environmental factors and positively correlated with 11 environmental factors, with 24.37% of the correlations being significant. MPA were negatively correlated with eight environmental factors and positively correlated with eight environmental factors, with all correlations being nonsignificant (Figure 5C).

Environmental factors were found to affect CH_4_ metabolic pathways, which explained 49.81% (RDA1) and 28.54% (RDA2) of the variation (Figure 5D). The CH_4_ uptake pathway was primarily negatively correlated with environmental factors, whereas the CH_4_ production pathway was primarily positively correlated with environmental factors. Specifically, compared with XuMP and RuMP pathways, the serine pathway was extremely sensitive to alterations in vegetation and environmental factors. The low proportion (12.5–18.7%) of significant correlations between the 16 environmental factors and CH_4_ production pathways is shown in Supplementary Table S3. Therefore, the CH_4_ production pathway was found to be more stable than the CH_4_ uptake pathway. Among the top five genes in terms of relative abundance, *mdh*, *ENO*, *ACSS*, and *glyA* were positively correlated with SBD, GSN, TN, and ST (Figure 5E; Supplementary Table S4).

**Figure 7:**
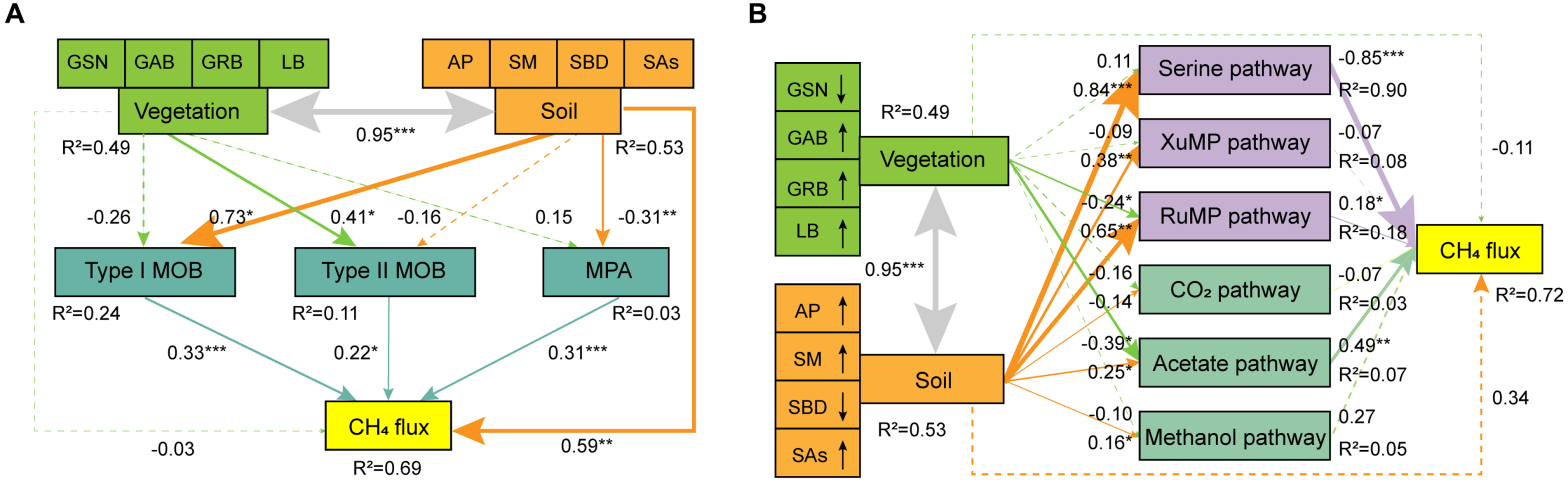
Response of CH_4_ flux to vegetation and soil factors. GE affects CH_4_ flux by altering different soil microbial taxa **(A)** and pathways **(B)**. The solid line indicates a significant level (*p* < 0.05), whereas the dotted line indicates a nonsignificant level. The size of the line indicates the value of the influence coefficient. *p < 0.05, **p < 0.01, ***p < 0.001. The line with an arrow indicates that the response of the indicator to grazing is increasing or decreasing in **(B)**. Global goodness of fit: Fisher’s C = 5.02, *p* = 0.081, AIC = 33.02, BIC = 72.05 in **(A)**; Fisher’s C = 11.37, *p* = 0.078, AIC = 49.37, BIC = 102.33 in **(B)**.

Finally, soil composite variables were established based on soil indicators (AP, SM, SBD, and SAs) using random forest analysis (Supplementary Figure S3), and the SEMs of vegetation, soil, microbial taxa, and CH_4_ metabolic pathways were established (Figure 7). Overall, the influence of soil on CH_4_ flux was more significant than that of vegetation. The type II MOB community was primarily affected by differences in vegetation, with a value of 0.41. Conversely, type I MOB and MPA communities were primarily affected by alterations in soil properties, with values of 0.73 and 0.31, respectively. CH_4_ flux was mainly regulated by serine and acetate pathways, with values of −0.85 and 0.79, respectively. The serine pathway was primarily driven by soil factors, whereas the acetate pathway was jointly driven by vegetation and soil factors. The SEMs revealed that high plant diversity and biomass were conducive for CH_4_ uptake. Conversely, compact and water-filled soil was conducive to CH_4_ production.

## 4 Discussion

### 4.1 Response of vegetation and soil features to GE

The effect of livestock on alpine meadows was determined via three mechanisms: trampling, defoliation (removal of plant shoot tissue), and returning of dung and urine (Liu et al., 2015). After removing grazers, higher contents of SOM, AN, AP, and AK were detected, possibly owing to the input of plant residues. The composition and transport of soil nitrogen alter methanotrophic activities (Jeremy et al., 2010). For example, ammonia nitrogen typically reduces CH_4_ uptake via product toxicity or competitive binding in CH_4_ monooxygenase (Nold et al., 1999; Jeremy et al., 2010). The physical properties of soil improved in terms of SBD and SAs (>1.0 mm; Table 1), which was conducive to vegetation growth (Li et al., 2018). In grassland ecosystems, herbage is an essential factor affecting CH_4_ metabolism, and CH_4_ fluxes in soil are altered by plant species and vegetation types (Praeg et al., 2017). Large herbivores promote the expansion of Gramineae forage by reducing the coverage of moss and shrubs via trampling and ingestion (Kitti et al., 2009; Falk et al., 2015). A high proportion of Leguminosae forage in a community increases CH_4_ emissions from shallow soil, whereas a high proportion of Gramineae forage decreases CH_4_ emissions (Khalsa et al., 2014). In the present study, GE significantly decreased the proportion of Gramineae forage but had no significant effect on Leguminosae forage (Figure 2). A high proportion of forbs, such as *Bistorta vivipara*, *Equisetum arvense*, and *Thalictrum alpinum*, was observed in GPs and NPs. Continuous high-intensity grazing increases the coverage of species that can adapt to drought habitats, such as *Plantago depressa* and *Leontopodium nanum*. However, it reduces the yield of high-quality forages, such as *Elymus dahuricus*, *E. nutans*, and *Elymus sibiricus*. GE can effectively reduce grassland degradation and accelerate ecological restoration. Reportedly, short-term GE can enhance plant diversity, whereas long-term GE can reduce species richness (Li et al., 2017), plant productivity, and soil organic carbon content (Cao et al., 2022). Fencing for 6 years may be adequate for vegetation and soil restoration (Li et al., 2018). Therefore, a 6-year GE period was selected in this study. However, fencing for 6 years reduced the potential of the CH_4_ sink in this study, and the optimal GE duration remains unclear. Short-term GE can enhance vegetation biomass, increase the proportion of large SAs, and reduce SBD. However, the effect of GE on vegetation characteristics tends to vary, and it is necessary to further distinguish the heterogeneous effects of GE on the vegetation of different patches in alpine regions.

### 4.2 Response of the methanotrophic community to GE

The amount of CH_4_ emissions from the soil to the atmosphere depends on the balance between methanotrophic and methanogenic communities. Our analyses of relative abundance and functional genes indicated that type II MOB were the dominant taxa involved in CH_4_ uptake in alpine meadow soil (Figures 3, 4). Conversely, type I MOB were the dominant taxa in wetlands, where *Methylomonas*, *Methylobacter*, and *Methylovulum* dominated the community (Liu et al., 2020). The MOB in sediment samples comprised *Methylococcus*, *Methylobacter*, and *Methylosinus* (type II MOB); type I MOB species are usually more abundant than type II MOB species (Yang et al., 2016). Our knowledge regarding metabolic versatility of type II MOB is expanding. For example, type II MOB were initially considered to have dedicated growth requirements. However, studies have shown that numerous species of type II MOB are parthenogenic and capable of growing in the presence of multicarbon compounds. For example, the strain SC2 can grow with mixed nutrients in the presence of H_2_ and CH_4_ (Myung et al., 2016; Wen et al., 2016; Hakobyan and Liesack, 2020). Further, *M. silvestris* can use various organic acids (succinate, pyruvate, malate, and acetate) and ethanol as the only growth substrates (Dedysh et al., 2005). Type II MOB exhibits distinct advantages over type I MOB, including a high acetyl coenzyme flux and the combination of two important greenhouse gasses (CH_4_ and CO_2_), and has been considered for industrial application (Nguyen et al., 2021). The findings of this study revealed that type I MOB can grow rapidly in a heterogeneous environment. However, type II MOB can adapt to more significant environmental fluctuations, especially in soils with high CH_4_ concentration (Macalady et al., 2002; Jeremy et al., 2010). Some studies have classified Types I and II MOB as R- and K-strategists, respectively (Steenbergh et al., 2009; Siljanen et al., 2011). ANME can proliferate in anoxic environments and drive the CH_4_ cycle in coastal sediments (Wallenius et al., 2021); however, they were not detected in this study. This may be related to the soil texture; SAs (>1.0 mm) accounted for 55.96–65.54% of the total particles, and the loose structure of soils facilitated oxygen flow. In addition, the ANME sequence library should be further expanded to ensure the accuracy of species annotation.

The dominant microbial taxa drive soil microbial functions (Jiao et al., 2022). Thus, understanding the composition of dominant species can help determine grassland CH_4_ sink and source transition. In the present study, the MOB genus *Methylocystis* exhibited the highest relative abundance. Some species were classified as facultative MOB because they used various carbon substrates, such as acetate and methanol. They grew in the temperature range of 15°C–37°C and pH range of 6.5–10. Their genome comprised two particulate CH_4_ monooxygenases (pMMOs), one for low-affinity CH_4_ oxidation and the other for high-affinity CH_4_ oxidation. Additionally, several genes are involved in nitrogen fixation, hydrogenase production, heavy metal resistance, and polyhydroxybutyrate synthesis (Jung et al., 2020). The diverse energy sources and metabolic versatility of type II MOB may explain their dominant CH_4_ uptake in alpine meadow soil. In grazing systems, MOB is a food source rich in K and contains certain trace elements, such as Mg and Fe (Kuzniar et al., 2019). From a thermodynamic perspective, the RuMP pathway is a more efficient carbon assimilation pathway than the serine pathway (Wang, 2020). Notably, almost all Gammaproteobacteria of MOB belong to the Methylococacaceae family. However, the *Crenothrix* genus belongs to the Crenotrichaceae family (Knief, 2015). Some cultivation-independent species remain poorly delineated, such as jasper ridge clusters (JR1, JR2, and JR3), upland soil clusters (USCα and USCγ), Moore house peat clusters, and rice paddy clusters. Thus, it is challenging to determine the exact number of known MOB at the species level because the taxonomic status of some species, such as *Methylococcus thermophilus*, *Methylococcus mobilis*, and *Methylococcus chroococcus*, remain unclear (Knief, 2015). Limited information on these uncultured methanotrophic and methanogenic bacteria restricts data interpretation.

### 4.3 Response of the methanogenic community to GE

GE increased the relative abundance of *Methanobrevibacter*, the dominant genus of MPA in alpine meadow soil. *Methanomethylovorans* is a widespread methanogenic archaeal genus that exists under freshwater conditions with a broad niche (Tsola et al., 2021). GE increased the GAB and SM, which decreased ST, consequently leading to the decline of *Methanosarcina* because the species in this genus are sensitive to temperature changes. A study reported that the relative abundance of *Methanosarcina* increased when the temperature increased from 10°C to 30°C (Chen et al., 2022). This increase may also be caused by alterations in substrates, such as acetate, CO_2_, and methanol, in the soil because *Methanosarcina* species are multitrophic methanogenic archaea that can use various simple compounds as substrates (Chen et al., 2022). Additionally, species such as *M. mazei*, *M. barkeri*, and *M. flavescens* were found only in NPs (Figure 4). The disappearance of archaeal species may be due to the removal of livestock excreta input, and methanogens are highly abundant in the intestines and feces of livestock (Vechi et al., 2022). However, the abundance of *M. millerae* (the absolute dominant species) increased by 219.05% in NPs compared to NPs. Thus, GE reduced the diversity of MPA but increased the relative abundance of the dominant methanogenic species.

This study assessed multiple functional genes together to analyze CH_4_ metabolic pathways. Previous studies have primarily used *pmoA* and *mcrA* to analyze microbial CH_4_ oxidation and production processes (Bhardwaj and Dubey, 2020; Zhang et al., 2022). *pmoA* is a common functional gene in MOB and is often used to assess the diversity and phylogeny of MOB in different environments (Pandit et al., 2016; Wen et al., 2016). The final step of methanogenesis is catalyzed by the protein encoded by *mcrA*, which is abundant in MPA and is considered a functional gene used to detect methanogenic community diversity (Harirchi et al., 2022; Shen et al., 2022). However, MOB detection using *pmoA* as a functional marker can reveal thousands of sequences representing unknown methanotrophic bacteria. This limits data interpretation as information regarding these uncultured MOB is limited. Some uncultured methanotrophs are considered vital for CH_4_ oxidation, although the biological mechanisms underlying the effects of other sequenced groups remain unclear (Knief, 2015). *pmoA* must be used cautiously when interpreting data regarding unknown MOB. In the present study, the acetate pathway was the dominant CH_4_ production pathway in terms of the relative abundance of related genes. *ACSS* alone catalyzes the conversion of acetate to acetyl-CoA, whereas *ackA* and *pta* together complete this process, thus increasing the extent of functional redundancy and improving the stability of the acetate pathway. *fwdA*, which catalyzes the production of formyl-MFR from CO_2_, a critical step in the acetate pathway, was found only in NPs.

### 4.4 CH_4_ metabolism and its driving factors

CH_4_ metabolism is regulated by various environmental factors (Wei et al., 2015; Liu et al., 2021), of which SM content and temperature are considered the dominant factors that control the CH_4_ cycle in grasslands (Wang et al., 2014). In the present study, AP, SAs, TP, GSN, and SM were the top five factors. The microbial community showed a positive response to soil phosphorus, especially under phosphorus-deficient soil conditions (Chen and Xiao, 2023); this is because soil phosphorus deficiency limits MOB growth (Sheng et al., 2016). Furthermore, GE increases AP content, thereby favoring the development of bacterial communities. The effect of SAs was second only to that of AP. Substantial interactions occur between microbial communities and soil properties during CH_4_ flux regulation (Sengupta and Dick, 2017; Zhang et al., 2022). Unlike MOB, MPA are specialized, strict anaerobes sensitive to oxygen. Thus, they are inhibited and sometimes killed under oxygen-rich conditions. MPA may grow gradually as they have limited available substrates and can only use simple substances, such as CO_2_, H_2_, formic acid, acetate, and methylamine. Such simple substances must be transformed by other fermentative bacteria, which break down complex organic matter and make it available to MPA (Chen et al., 2022). GE can indeed improve the physical properties of soil (SBD, SAs, and SM), which impact microbial communities and CH_4_ flux diffusivity. In addition, this study examined the response of three methanogenic pathways in the KEGG database to grazing exclusion. Previous studies have shown that methanogens are classified into three groups, including hydrogenotrophic, aceticlastic and methylotrophic, based on their substrate utilization (Lyu et al., 2018). Different classification categories may lead to different results, but the response of dominant species to environmental factors is certain, and focusing on changes in dominant species may be crucial to explaining CH_4_-oxidation.

Notably, MOB and MPA exhibited heterogeneous responses to environmental factors in this study. Type I MOB were primarily affected by SM and SBD, whereas type II methanotrophs were primarily affected by TP, SBD, and SM. *Microvirga ossetica*, *M. extorquens*, *M. rosea*, *M. populi*, and *P. koreensis* belong to MOB. *M. ossetica* and *M. rosea* were positively and negatively affected by AP, respectively. Therefore, changes in AP cannot be interpreted only as a decrease or increase in the CH_4_ uptake potential. The insensitivity of *M. populi* and *P. koreensis* to environmental factors increases the stability of the MOB community under different environmental conditions. A high proportion of species in type I MOB varied synergistically with the environment, whereas an even higher proportion of species in type II MOB and MPA varied antagonistically with the environment. Thus, type I MOB were more susceptible to environmental impacts than type II MOB and MPA. When the feedback of species to environmental factors was diverse, the redundancy of community functions increased.

Large herbivores are additional factors that may substantially alter high-latitude landscape characteristics. However, their potential impact is rarely considered in the alpine ecosystem (Fischer et al., 2022). The aboveground biomass in fenced meadows was higher than that in grazing meadows, i.e., the abundance of Leguminosae and Poaceae forage increased and that of Cyperaceae forage decreased due to grazing (Li et al., 2019). Regarding belowground ecosystem properties, studies have shown that changes in ST, moisture, and texture are associated with disturbances caused by large animals (Olofsson et al., 2001; Beer et al., 2020). However, forbs, including *B. vivipara*, *E. arvense*, and *T. alpinum*, were the dominant species (78.9% in GPs and 72.1% in NPs) in the experimental area in this study. Nevertheless, there is a lack of reference data regarding CH_4_ uptake and production from forb patches and their responses to GE. In the present study, alpine meadow soil was a CH_4_ sink (including GPs and NPs in the growing season) whose potential was reduced by GE. However, SEMs revealed that the influence of soil (especially soil physical factors) on CH_4_ flux was greater than that of vegetation. Moreover, an increase in SM negatively affects aerobic MOB, and a decrease in plant diversity may promote this trend. CH_4_ can be produced not only by microorganisms (microbial CH_4_) but also by natural gas sealed under or inside the permafrost that is emitted during thawing (thermogenic CH_4_) (Froitzheim et al., 2021). During autumn and winter, the primary source of CH_4_ is thermogenic CH_4_, and increased CH_4_ concentration associated with microbial processes is observed during spring and summer (Li et al., 2022). The CH_4_ produced by abiotic factors and different lifestyles of plants should be investigated in studies on CH_4_ flux, and their changes should be monitored in different seasons to refine CH_4_ studies in alpine meadow ecosystems.

## 5 Conclusion

GE significantly altered the vegetation structure, increasing the importance value of Cyperaceae while decreasing the importance value of Graminaea, and improved the physical properties and nutrient content of the soil. Alpine meadow soil is a CH_4_ sink; type II MOB were the dominant taxa involved in CH_4_ uptake, with the representative group of *Methylocistis*. The diversity and abundance of MPA were significantly lower than those of MOB, with the representative group of *Methanobrevibacter*. *M. aurea*, *M. silvestris*, *M. bryophila*, and *M. trichosporium* were the biomarkers in NPs, whereas *M. rosea*, *M. mazei*, *M.* sp. SC2, *M. luteus*, and *M. tundripaludum* were the biomarkers in GPs. CH_4_ flux was mainly regulated by serine and acetate pathways, and the serine pathway was primarily driven by soil factors, whereas the acetate pathway was mainly driven by soil and vegetation factors. The influence of soil on methanotrophic and methanogenic microbial taxa was greater than that of vegetation. Type II MOB and MPA taxa were more stable during the response to environmental changes than type I MOB. Regarding CH_4_ production, GE reduced the diversity of MPA. However, the relative abundance of *M. millerae* (the absolute dominant species in the methanogenic community) increased substantially. Our study shows that GE increases soil CH_4_ flux and alters methanotrophic and methanogenic communities during the growing season in alpine meadows. However, the functions of soil microbial communities are usually determined by dominant taxa, and further studies are needed to characterize the biology of *Methylocystis* and *Methanobrevibacter*, the dominant genus in alpine meadow soils, and their responses to environmental factors.

## Data availability statement

The datasets presented in this study can be found in online repositories. The names of the repository/repositories and accession number(s) can be found at: https://www.ncbi.nlm.nih.gov/, PRJNA837727.

## Author contributions

SW: Conceptualization, Data curation, Funding acquisition, Methodology, Software, Writing – original draft. XC: Investigation, Software, Writing – review & editing. WL: Investigation, Writing – review & editing. WG: Investigation, Methodology, Writing – review & editing. ZW: Software, Writing – review & editing. WC: Data curation, Funding acquisition, Methodology, Software, Writing – review & editing.

## Footnotes

^1^ https://www.bioincloud.tech/standalone-task-ui/network
