# Supplementary material for: Grazing exclusion alters soil methane flux and methanotrophic and methanogenic communities in alpine meadows on the Qinghai–Tibet Plateau
Source: Front Microbiol. 2023 Dec 18;14:1293720. doi: 10.3389/fmicb.2023.1293720 (PMC10757936; doi:10.3389/fmicb.2023.1293720)
Supplement: Supplementary file 1 [file Data_Sheet_1.PDF]

Supplementary Material

1.1 Supplementary Figures

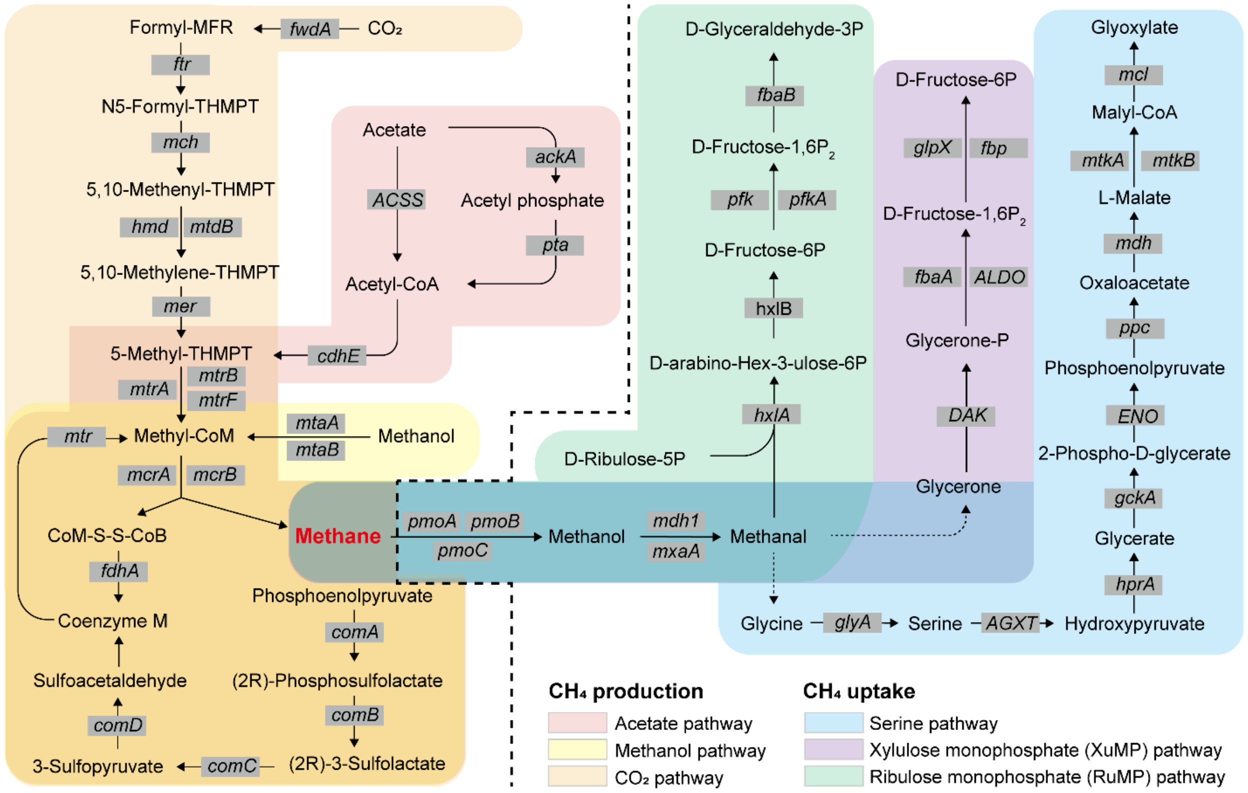

Supplementary Figure 1. Partial pathway of microbial CH_4 metabolism in soil. The different colored modules indicate the pathways for CH_4 uptake and production.

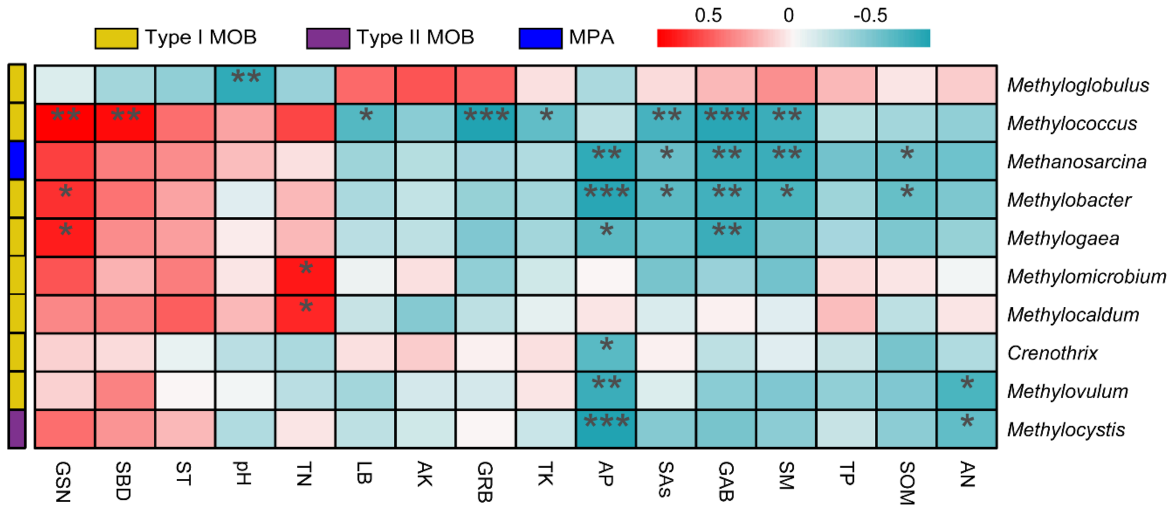

Supplementary Figure 2. Correlation analysis including MOB and MPA at the genus level and environmental factors. Features with no significant correlation were removed. Abbreviations: GSN, grass species number; GAB, grass aboveground biomass; GRB, grass root biomass; LB, litter biomass; SBD, soil bulk density; SAs, soil aggregates; ST, soil temperature; SM, soil moisture; SOM, soil organic matter; AN, available nitrogen; AP, available phosphorus; AK, available potassium; TN, total nitrogen; TP, total phosphorus; TK, total potassium; MOB, methane-oxidizing bacteria; MPA, methane-producing archaea. $^*P < 0.05$, ** $P < 0.01$, *** $P < 0.001$.

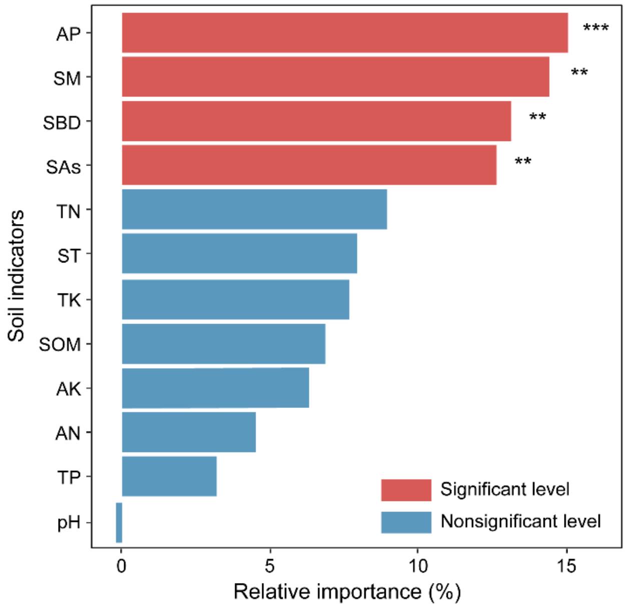

Supplementary Figure 3. Relative importance of soil indicators for CH_4 flux using random forest analysis. ** $P < 0.01$, *** $P < 0.001$.

1.2 Supplementary Tables

Supplementary Table 1. Processing and quality control of raw sequencing data.

Sample ID	Raw reads	Raw base (GB)	Raw Q20 (%)	Raw Q30 (%)	Clean reads	Cleaned (%)	Clean Q20 (%)	Clean Q30 (%)
GP1	35386227	10.62	97.66	93.95	33789874	95.49	98.88	95.90
GP2	33591932	10.08	97.67	93.96	32126869	95.64	98.88	95.90
GP3	33634563	10.09	97.86	94.29	32228860	95.82	98.93	96.02
GP4	38986060	11.70	97.75	94.11	37311584	95.70	98.90	95.97
GP5	45461699	13.64	97.73	94.09	43409906	95.49	98.91	96.00
GP6	33590706	10.08	97.60	93.78	32009145	95.29	98.83	95.77
NP1	32993232	9.90	97.77	94.28	31680301	96.02	98.95	96.15
NP2	34399624	10.32	97.52	93.77	32793301	95.33	98.86	95.90
NP3	34434125	10.33	97.59	93.89	32833412	95.35	98.89	95.97
NP4	33159770	9.95	97.57	93.70	31763245	95.79	98.80	95.65
NP5	37238088	11.17	97.65	93.99	35579040	95.54	98.90	95.99
NP6	34626565	10.39	97.58	93.87	33070989	95.51	98.88	95.93

GP: grazed plot, NP: nongrazed plot. Q20: the proportion of bases with a quality score > 20; Q30: the percentage of bases with a quality score > 30. Clean reads: the number of reads after quality control and host-removing. Cleaned (%): the proportion of remaining sequences after filtering.

Supplementary Table 2. Plant species composition and its importance values in the experimental site.

No.	Family	Genus	Species	Importance values (%)	
				Grazed plots	Nongrazed plots
1	Polygonaceae	*Bistorta*	*Bistorta vivipara*	11.43±2.88	7.67±0.73**
2	Equisetaceae	*Equisetum*	*Equisetum arvense*	1.84±0.42	13.89±2.38***
3	Ranunculaceae	*Thalictrum*	*Thalictrum alpinum*	5.53±2.45	5.95±3.38
4	Cyperaceae	*Carex*	*Carex atrofusca*	—	9.99±1.24
5	Primulaceae	*Lysimachia*	*Lysimachia maritima*	4.41±2.38	2.95±0.68
6	Gramineae	*Achnatherum*	*Achnatherum inebrians*	6.74±3.20	—
7	Leguminosae	*Medicago*	*Medicago ruthenica*	1.73±0.93	4.23±2.12
8	Gramineae	*Poa*	*Poa araratica*	3.53±0.88	2.24±1.36
9	Rosaceae	*Argentina*	*Argentina anserina*	2.35±1.05	5.47±0.62**
10	Orobanchaceae	*Euphrasia*	*Euphrasia pectinata*	0.73±0.69	4.67±1.25
11	Plantaginaceae	*Plantago*	*Plantago depressa*	4.65±0.34	0.48±0.38**
12	Rosaceae	*Sibbaldianthe*	*Sibbaldianthe bifurca*	4.86±2.76	—
13	Apiaceae	*Carum*	*Carum carvi*	4.48±2.26	—
14	Mazaceae	*Lancea*	*Lancea tibetica*	2.78±0.30	1.65±1.65
15	Cyperaceae	*Carex*	*Carex capillifolia*	1.44±1.23	2.97±1.85**
16	Gentianaceae	*Gentianopsis*	*Gentianopsis paludosa*	—	4.28±1.51
17	Gentianaceae	*Lomatogonium*	*Lomatogonium carinthiacum*	—	4.23±0.87
18	Apiaceae	*Pleurospermum*	*Pleurospermum uralense*	3.54±1.41	0.36±0.36
19	Gramineae	*Elymus*	*Elymus nutans*	1.19±0.66	2.19±1.15
20	Compositae	*Leontopodium*	*Leontopodium nanum*	3.28±1.69	—
21	Ranunculaceae	*Thalictrum*	*Thalictrum aquilegiifolium* var. *sibiricum*	1.77±1.77	1.47±1.45
22	Compositae	*Saussurea*	*Saussurea epilobioides*	0.50±0.50	2.54±0.92*
23	Ranunculaceae	*Ranunculus*	*Ranunculus tanguticus*	0.88±0.75	2.13±1.64
24	Polygonaceae	*Persicaria*	*Persicaria taquetii*	0.67±0.67	2.27±1.13*
25	Polygonaceae	*Bistorta*	*Bistorta macrophylla*	1.46±1.34	1.29±0.65
26	Compositae	*Leontopodium*	*Leontopodium haplophylloides*	0.83±0.83	1.77±0.85**
27	Leguminosae	*Oxytropis*	*Oxytropis ochrocephala*	1.79±1.79	0.70±0.70
28	Leguminosae	*Astragalus*	*Astragalus przewalskii*	2.46±1.25	—
29	Compositae	*Artemisia*	*Artemisia smithii*	2.30±0.99	—
30	Gramineae	*Elymus*	*Elymus sibiricus*	0.35±0.35	1.87±1.87
31	Compositae	*Taraxacum*	*Taraxacum mongolicum*	1.53±0.75	0.65±0.39
32	Cyperaceae	*Carex*	*Carex alatauensis*	1.37±1.37	0.79±0.79**
33	Scrophulariaceae	*Pedicularis*	*Pedicularis kansuensis*	1.70±0.79	0.37±0.37
34	Plantaginaceae	*Plantago*	*Plantago asiatica*	1.73±0.93	0.34±0.34
35	Rosaceae	*Potentilla*	*Potentilla multifida*	0.35±0.35	1.70±1.70*
36	Gramineae	*Stipa*	*Stipa capillacea*	1.99±1.49	—
37	Gentianaceae	*Swertia*	*Swertia tetraptera*	—	1.98±1.58
38	Gramineae	*Elymus*	*Elymus dahuricus*	0.35±0.35	1.53±0.76*

39	Cyperaceae	*Carex*	*Carex lehmannii*	—	1.83±0.85
40	Cyperaceae	*Blysmus*	*Blysmus sinocompressus*	—	1.82±0.97
41	Compositae	*Aster*	*Aster altaicus*	1.77±1.77	—
42	Compositae	*Artemisia*	*Artemisia phaeolepis*	1.73±1.24	—
43	Gramineae	*Poa*	*Poa pratensis*	1.41±1.37	0.27±0.16
44	Gramineae	*Poa*	*Poa albertii*	0.83±0.83	0.77±0.77
45	Leguminosae	*Cardamine*	*Cardamine tangutorum*	0.83±0.83	0.71±0.71
46	Rosaceae	*Potentilla*	*potentilla discolor*	1.45±1.45	—
47	Compositae	*Aster*	*Aster semiprostratus*	1.42±0.71	—
48	Geraniaceae	*Geranium*	*Geranium sibiricum*	1.41±1.48	
49	Caryophyllaceae	*Stellaria*	*Stellaria media*	0.87±0.87	0.51±0.28
50	Compositae	*Heteropappus*	*Heteropappus altaicus*	1.38±1.38	—
51	Compositae	*Saussurea*	*Saussurea katochaete*	1.33±1.33	—
52	Celastraceae	*Parnassia*	*Parnassia trinervis*	—	1.18±0.68
53	Orobanchaceae	*Pedicularis*	*Pedicularis longiflora*	0.86±0.86	—
54	Gentianaceae	*Gentiana*	*Gentiana lawrencei* var. *farreri*	0.68±0.68	0.17±0.17
55	Gentianaceae	*Gentiana*	*Gentiana straminea*	0.79±0.79	—
56	Gentianaceae	*Gentiana*	*Gentiana squarrosa*	0.35±0.35	0.42±0.22
57	Cyperaceae	*Carex*	*Carex parvula*	0.75±0.75	—
58	Compositae	*Aster*	*Aster alpinus*	0.70±0.70	—
59	Scrophulariaceae	*Pedicularis*	*Pedicularis flava*	0.55±0.36	0.13±0.13
60	Plumbaginaceae	*Plumbagella*	*Plumbagella micrantha*	0.53±0.35	—
61	Violaceae	*Viola*	*Viola biflora*	0.53±0.39	—
62	Violaceae	*Viola*	*Viola prionantha*	0.50±0.50	—
63	Caryophyllaceae	*Silene*	*Silene gallica*	—	0.50±0.50
64	Papaveraceae	*Hypecoum*	*Hypecoum leptocarpum*	0.50±0.50	—
65	Cyperaceae	*Carex*	*Carex pseuduncinoides*	0.40±0.40	—
66	Primulaceae	*Primula*	*Primula nutans*	0.20±0.20	0.19±0.09
67	Polygonaceae	*Rumex*	*Rumex crispus*	—	0.38±0.38
68	Leguminosae	*Astragalus*	*Astragalus polycladus*	0.34±0.34	—
69	Leguminosae	*Tibetia*	*Tibetia himalaica*	0.34±0.34	—
70	Ranunculaceae	*Trollius*	*Trollius chinensis*	—	0.27±0.16
71	Ranunculaceae	*Anemone*	*Anemone cathayensis*	—	0.23±0.18
72	Gentianaceae	*Gentiana*	*Gentiana officinalis*	0.12±0.12	—
73	Rubiaceae	*Galium*	*Galium spurium*	—	0.17±0.11

* $P < 0.05$, ** $P < 0.01$, *** $P < 0.001$.

Supplementary Table 3. Correlation analysis including methanotrophic and methanogenic pathways and environmental factors.

Environmental factors	CH_4 uptake			CH_4 production		
	Serine pathway	XuMP pathway	RuMP pathway	CO_2 pathway	Acetate pathway	Methanol pathway
GSN	0.83**	0.04	0.36	-0.30	-0.06	-0.28
GAB	-0.10**	-0.32	-0.45	0.24	0.15	0.27

GRB	-0.80**	-0.34*	-0.44	-0.10	-0.28*	-0.08
LB	-0.91**	-0.24	-0.37	0.16	0.09	0.18
SBD	0.86**	0.12	0.29*	-0.02	0.06	-0.04*
SAs	-0.78**	-0.08	-0.47*	0.42*	0.14	0.39
ST	0.57	-0.14	-0.02	-0.05	-0.11	-0.07
SM	-0.96**	-0.35**	-0.48	0.25	0.16	0.28*
pH	0.16	0.05	0.16	0.29	-0.04	0.30
SOM	-0.62*	-0.42	-0.04	0.29	0.07	0.34
AN	-0.60*	-0.30	-0.00	0.22	0.25	0.32
AP	-0.62*	-0.60*	-0.34**	0.65*	0.52*	0.70*
AK	-0.61*	0.11	0.03	0.05	0.12	0.04
TN	0.42	-0.29	0.04	0.15	0.01	0.13
TP	-0.53	-0.61	-0.27	0.10	-0.14	0.13
TK	-0.58*	0.11	-0.56	0.41	0.11	0.31

Abbreviations: GSN, grass species number; GAB, grass aboveground biomass; GRB, grass root biomass; LB, litter biomass; SBD, soil bulk density; SAs, soil aggregates; ST, soil temperature; SM, soil moisture; SOM, soil organic matter; AN, available nitrogen; AP, available phosphorus; AK, available potassium; TN, total nitrogen; TP, total phosphorus; TK, total potassium; MOB, methane-oxidizing bacteria; MPA, methane-producing archaea. *$P < 0.05$, ** $P < 0.01$, *** $P < 0.001$.

Supplementary Table 4. Correlation analysis including functional genes and environmental factors.

Functional genes	GSN	GAB	GRB	LB	SBD	SAs	ST	SM	pH	SOM	AN	AP	AK	TN	TP	TK
hprA	-0.71	0.55	0.27	0.49	-0.47	0.49	-0.56	0.51	0.26	0.74*	0.45	0.47	0.38	-0.43	0.14	0.29
mdh	0.19	-0.17	-0.52	-0.27	0.43	-0.23	0.17	-0.19	0.13	-0.01	0.13	0.39	-0.22	0.27	-0.29	-0.15
glyA	0.70*	-0.80**	-0.63*	-0.61*	0.42	-0.71**	0.38	-0.80**	0.32	-0.45	-0.50	-0.71*	-0.35	0.04	-0.55	-0.56
AGXT	0.12	-0.20	-0.00	-0.10	-0.12	-0.20	0.19	-0.21	0.19	-0.42	-0.35	-0.50	-0.08	-0.37	-0.69*	0.19
ppc	0.07	-0.34	-0.28	-0.25	0.10	-0.09	-0.20	-0.33	-0.25	-0.49	-0.66*	-0.73**	0.06	-0.49	-0.67*	0.11
ENO	0.57	-0.74**	-0.48	-0.65*	0.56	-0.46	0.36	-0.72**	-0.03	-0.71**	-0.69*	-0.83**	-0.48	0.15	-0.28	-0.36
mcl	0.57	-0.58*	-0.43	-0.56	0.47	-0.68*	0.31	-0.63*	-0.06	-0.38	-0.31	-0.52	-0.08	0.20	-0.59*	-0.45
mtkB	0.36	-0.21	0.03	-0.29	0.42	-0.28	0.42	-0.23	-0.27	0.09	-0.09	0.09	-0.13	0.78**	0.29	-0.14
gckA	-0.55	0.40	0.06	0.36	-0.26	0.45	-0.60*	0.37	0.18	0.43	0.64*	0.33	0.29	-0.24	0.44	-0.02
mtkA	-0.26	0.26	-0.05	0.11	0.11	0.21	-0.16	0.21	-0.24	0.24	0.32	0.47	0.31	0.30	0.08	0.25
DAK	-0.36	0.33	0.09	0.22	-0.13	0.08	-0.60*	0.29	-0.13	0.58	0.58*	0.34	0.33	-0.23	0.24	-0.32
fbp	0.22	-0.07	0.19	0.02	-0.18	-0.19	0.17	-0.06	0.03	-0.10	0.01	-0.22	0.23	0.01	-0.17	-0.06
ALDO	0.05	-0.18	-0.36	-0.14	0.14	-0.04	-0.28	-0.14	-0.11	-0.19	-0.18	-0.20	-0.17	-0.38	-0.20	-0.21
fbaA	-0.07	-0.24	-0.20	-0.13	-0.03	-0.01	-0.30	-0.26	-0.09	-0.43	-0.38	-0.75**	0.21	-0.50	-0.55	0.11
glpX	0.25	-0.42	-0.65*	-0.48	0.60*	-0.15	0.30	-0.45	0.14	-0.36	-0.25	-0.00	-0.25	0.38	-0.50	0.16
fbp-SEBP	0.20	-0.36	-0.31	-0.24	0.02	-0.33	-0.03	-0.39	0.20	-0.32	-0.28	-0.58	0.06	-0.41	-0.72**	-0.15
glpX-SEBP	-0.35	0.35	0.25	0.36	-0.38	0.17	-0.13	0.29	0.22	0.37	0.37	0.28	0.34	-0.13	-0.09	0.35
fbaB	-0.43	0.53	0.29	0.56	-0.42	0.47	-0.19	0.49	-0.20	0.14	0.48	0.34	0.56	0.08	0.24	0.62*
pfkA	-0.15	0.26	-0.19	0.37	-0.33	0.00	-0.51	0.22	-0.05	0.38	0.54	0.26	0.56	-0.35	0.01	-0.25
hxlA	-0.30	0.60*	0.29	0.66*	-0.50	0.31	-0.36	0.58*	-0.37	0.50	0.71**	0.58	0.70*	0.05	0.46	0.25
pfk	0.18	-0.29	-0.18	-0.15	-0.03	-0.34	-0.15	-0.33	-0.00	-0.18	-0.14	-0.63*	0.27	-0.28	-0.41	-0.29
pmoA	0.39	-0.45	-0.31	-0.57	0.56	-0.48	0.46	-0.46	0.52	0.05	-0.03	0.10	-0.63*	0.42	-0.12	-0.50
pmoB	-0.29	0.27	0.34	0.09	0.01	0.48	0.06	0.31	0.03	0.17	0.13	0.49	-0.03	0.32	0.29	0.49
pmoC	0.52	-0.60*	-0.48	-0.73**	0.81**	-0.40	0.51	-0.57	0.33	-0.13	-0.22	0.07	-0.76**	0.64*	0.04	-0.48
mdh1	-0.48	0.27	0.43	0.20	-0.29	0.49	-0.06	0.30	0.40	0.25	-0.15	0.17	-0.13	-0.24	0.10	0.49
fwdA	-0.57	0.40	0.26	0.42	-0.47	0.54	-0.18	0.39	0.43	0.30	0.07	0.30	0.18	-0.33	-0.02	0.63*
mer	0.23	-0.58*	-0.47	-0.45	0.31	-0.23	-0.08	-0.59*	-0.00	-0.55	-0.63*	-0.90**	-0.14	-0.26	-0.45	-0.18
ftr	-0.00	-0.31	-0.34	-0.25	0.28	0.08	-0.01	-0.32	0.40	-0.17	-0.08	-0.31	-0.32	0.12	0.29	-0.20
mtdB	0.29	-0.28	-0.24	-0.43	0.60*	-0.12	0.43	-0.29	-0.13	-0.12	-0.18	0.20	-0.20	0.79**	-0.01	0.12
mch	-0.21	0.37	0.27	0.30	-0.10	0.38	0.08	0.37	-0.35	0.11	0.18	0.47	0.31	0.43	0.29	0.60*
cdhE	0.35	-0.30	-0.02	-0.19	-0.02	-0.49	0.21	-0.34	0.13	-0.03	-0.17	-0.42	0.18	0.01	-0.38	-0.30
pta	-0.49	0.74**	0.50	0.70*	-0.58*	0.41	-0.61*	0.74**	-0.77**	0.24	0.55	0.31	0.68*	-0.37	0.33	0.25
ackA	0.47	-0.52	-0.51	-0.48	0.38	-0.44	0.18	-0.49	0.11	-0.31	-0.50	-0.32	-0.35	-0.12	-0.48	-0.44
ACSS	0.58*	-0.57	-0.76**	-0.56	0.56	-0.50	0.32	-0.57	0.08	-0.50	-0.30	-0.18	-0.29	0.14	-0.66*	-0.34

mtaB	-0.54	0.59*	0.30	0.57	-0.47	0.65*	-0.34	0.60*	0.04	0.28	0.54	0.56	0.44	-0.13	0.27	0.57
fdhA	0.16	0.23	0.12	0.11	0.05	-0.13	0.17	0.24	-0.30	0.36	0.34	0.70*	0.11	0.41	0.20	-0.10
mtr	-0.65*	0.48	0.09	0.50	-0.47	0.62*	-0.56	0.46	0.34	0.34	0.51	0.35	0.40	-0.42	0.19	0.34
mtrF	0.35	-0.30	-0.02	-0.19	-0.02	-0.49	0.21	-0.34	0.13	-0.03	-0.17	-0.42	0.18	0.01	-0.38	-0.30
mcr	-0.15	0.20	0.33	0.13	-0.10	0.46	0.12	0.27	-0.08	-0.24	-0.04	0.18	-0.08	0.09	0.11	0.56
comC	-0.36	0.33	0.09	0.22	-0.13	0.08	-0.60*	0.29	-0.13	0.58	0.58*	0.34	0.33	-0.23	0.24	-0.32
comD	0.14	-0.16	-0.18	-0.28	0.39	0.02	0.47	-0.13	0.34	0.04	-0.18	0.44	-0.51	0.49	0.03	0.16
mtrB	0.38	-0.36	-0.24	-0.47	0.59*	-0.23	0.48	-0.37	-0.10	-0.12	-0.22	0.10	-0.15	0.78**	-0.10	0.05
mcrA	-0.12	0.31	0.33	0.30	-0.27	0.37	-0.01	0.37	-0.13	-0.13	0.26	0.22	0.13	0.01	0.25	0.33
mcrB	0.05	-0.31	-0.34	-0.26	0.21	-0.00	-0.24	-0.28	-0.40	-0.57	-0.64*	-0.65*	-0.06	-0.43	-0.53	0.13
mtrA	0.24	-0.27	-0.01	-0.23	0.04	-0.34	0.12	-0.28	0.26	-0.10	-0.05	-0.33	-0.10	-0.12	-0.32	-0.31
comA	-0.68*	0.80**	0.45	0.83**	-0.73**	0.62*	-0.56	0.78**	-0.39	0.33	0.49	0.44	0.69*	-0.44	0.17	0.67*
mtrD	0.70*	-0.75**	-0.54	-0.78**	0.80**	-0.58	0.58*	-0.72**	0.09	-0.32	-0.62*	-0.27	-0.64*	0.54	-0.20	-0.47

Abbreviation: GSN, grass species number; GAB, grass aboveground biomass; GRB, grass root biomass; LB, litter biomass; SBD, soil bulk density; SAs, soil aggregates; ST, soil temperature; SM, soil moisture; SOM, soil organic matter; AN, soil available nitrogen; AP, available phosphorus; AK, available potassium; TN, total nitrogen; TP, total phosphorus; TK, total potassium. *$P < 0.05$, ** $P < 0.01$, *** $P < 0.001$.
